# The homology of odontodes in gnathostomes: insights from *Dlx *gene expression in the dogfish, *Scyliorhinus canicula*

**DOI:** 10.1186/1471-2148-11-307

**Published:** 2011-10-18

**Authors:** Mélanie Debiais-Thibaud, Silvan Oulion, Franck Bourrat, Patrick Laurenti, Didier Casane, Véronique Borday-Birraux

**Affiliations:** 1 Evolution des familles multigéniques, Laboratoire Evolution Génome et Spéciation, UPR9034 CNRS, 1 avenue de la terrasse, 91198 Gif-sur-Yvette, France; 2 UFR des Sciences du Vivant, Université Paris Diderot Sorbonne Paris Cité, 5 rue Marie-Andrée Lagroua Weill-Hallé, 75205 Paris Cedex 13, France; 3 UFR Sciences, Université Paris-Sud 11, 91405 Orsay Cedex, France; 4 Laboratoire Neurobiologie et Développement, Institut de Neurobiologie Alfred Fessard, UPR3294 CNRS, 1 avenue de la terrasse, 91198 Gif-sur-Yvette, France; 5 Département Forme, Institut des Sciences de l'Evolution - Montpellier, UMR5554 CNRS/Université Montpellier II, Place Eugène Bataillon, 34095 Montpellier cedex05, France; 6 Evolution et développement des chordés, Biologie Intégrative des Organismes Marins, UMR7232 CNRS/UMPC Université Paris 6, Observatoire océanologique, Avenue du Fontaulé, 66650 Banyuls-sur-Mer, France

## Abstract

**Background:**

Teeth and tooth-like structures, together named odontodes, are repeated organs thought to share a common evolutionary origin. These structures can be found in gnathostomes at different locations along the body: oral teeth in the jaws, teeth and denticles in the oral-pharyngeal cavity, and dermal denticles on elasmobranch skin. We, and other colleagues, had previously shown that teeth in any location were serially homologous because: i) pharyngeal and oral teeth develop through a common developmental module; and ii) the expression patterns of the *Dlx *genes during odontogenesis were highly divergent between species but almost identical between oral and pharyngeal dentitions within the same species. Here we examine *Dlx *gene expression in oral teeth and dermal denticles in order to test the hypothesis of serial homology between these odontodes.

**Results:**

We present a detailed comparison of the first developing teeth and dermal denticles (caudal primary scales) of the dogfish (*Scyliorhinus canicula*) and show that both odontodes develop through identical stages that correspond to the common stages of oral and pharyngeal odontogenesis. We identified six *Dlx *paralogs in the dogfish and found that three showed strong transcription in teeth and dermal denticles (*Dlx3*, *Dlx4 *and *Dlx5*) whereas a weak expression was detected for *Dlx1 *in dermal denticles and teeth, and for *Dlx2 *in dermal denticles. Very few differences in *Dlx *expression patterns could be detected between tooth and dermal denticle development, except for the absence of *Dlx2 *expression in teeth.

**Conclusions:**

Taken together, our histological and expression data strongly suggest that teeth and dermal denticles develop from the same developmental module and under the control of the same set of *Dlx *genes. Teeth and dermal denticles should therefore be considered as serial homologs developing through the initiation of a common gene regulatory network (GRN) at several body locations. This mechanism of heterotopy supports the 'inside and out' model that has been recently proposed for odontode evolution.

## Background

Teeth and tooth-like structures, together designated as odontodes, are thought to be serial homologs: they are repeated mineralized units composed of dentine and enamel, or enameloid, surrounding a pulp cavity [[1,2], see for review: [3]]. Odontodes can be found in various locations on the body of extant gnathostomes, such as teeth in jaws and different bones in the oral and pharyngeal cavity, but also as dermal denticles (also called placoid scales) on the body surface in chondrichthyans [4]. Teeth (oral or pharyngeal) contrast with denticles (pharyngeal or dermal) in their ability to regenerate through a typical renewing process [5]. There has been a long and recently revitalized debate concerning the origin and evolution of odontodes. Due to their mineralized composition, they are well preserved in the fossil record and a diversity of odontodes has been described in fossil and extant taxa belonging to gnathostomes: dermal denticles in thelodonts or heterostracans, pharyngeal denticles/teeth in thelodonts, ornaments on dermal bones of placoderms or coelacanths, or the earliest oral teeth described in placoderms [6]. More controversial are the pharyngeal denticles/teeth found in conodont animals that are currently considered to have diverged early from other vertebrates [7,8]. The long-held view [1] that oral teeth first evolved by the co-option of dermal denticles at the oral margin when jaws evolved (the outside-in hypothesis) has been challenged by reconsideration of these fossil data. Because pharyngeal denticles may have arisen before oral teeth and because both structures share a common organization, Smith and Coates [9,10] favoured a recruitment of the gene regulatory network (GRN) responsible for pharyngeal teeth development from the pharynx to the jaw in early gnathostomes (the inside-out hypothesis). This model has been supported by morphological and molecular data gained in teleosts: pharyngeal tooth development has been compared to that of the mouse oral teeth, showing that similar signalling and transcription factors are expressed during oral and pharyngeal odontogenesis [11-13]. However, detailed comparison of expression patterns between zebrafish pharyngeal and mouse oral tooth development showed differences and some molecular markers are specific for mouse oral (*Pax9 *[13,14]) or zebrafish pharyngeal (*eve1 *[15]) odontogenesis. Additional studies have focused on comparative analysis between oral and pharyngeal dentitions within a given organism. They showed that, in extant teleost fish, teeth in the jaw or in the pharynx develop through similar gene expression patterns [11,16-18]. These results support the hypothesis that a single developmental GRN is initiated at different locations to make up oral and pharyngeal teeth through a simple mechanism of heterotopy [18]. These studies led to a more comprehensive scenario about odontode origin and evolution (named the "inside and out" model) that postulates serial homology between all gnathostome odontodes, as defined by the sharing of a common GRN for their development [19]. Oral teeth, pharyngeal teeth/denticles, and dermal denticles would then belong to this odontode group, developmentally characterized by: (i) the presence of a neural-crest derived mesenchyme; (ii) any epithelium able to respond to a mesenchyme signal [19].

In order to test the "inside and out" model, we searched for both developmental and genetic similarities between dermal denticles and oral teeth in the dogfish, *Scyliorhinus canicula*. In this species, histological observations support that tooth and dermal denticle development display similarities with that of osteichthyans [20,21]. Among the different subsets of dermal denticle described during dogfish embryogenesis by Mellinger and Wrisez [22], we chose to work on the earliest developing ones, the caudal primary scales. They are located at the very tip of the tail, develop from caudal to rostral, are clearly observable in 28 mm long embryos, and are organised as four bilateral lines (two dorsal and two ventral lines with usually ten and eight scales respectively). Currently, only few expression data have been described for tooth and dermal denticles development in chondrichthyans: *Shh *[23], *Epha4 *[24], *Runx1 *and *Runx3 *[25], each gene showing a similar expression pattern in both structures. These expression patterns were not characterized on histological sections, therefore the tissue-specific transcriptional dynamics (epithelium *vs *mesenchyme) cannot be compared between structures or to other gnathostome species. To test the hypothesis of serial homology between tooth and dermal denticle development, we have characterised the expression of all *Dlx *gene family members identified in the dogfish, following a strategy that already allowed us to propose that a single GRN is involved in both oral and pharyngeal teeth in medaka [18]. This gene family represents a paradigmatic genetic marker to test if one or two independent GRN are involved in tooth-like structures because: (i) this gene family includes at least six members in gnathostomes, transcribed with different expression patterns during tooth development in the mouse [26,27] and teleosts [12,18,28], and (ii) contrary to the variability of *Dlx *patterns between species, the regulation of *Dlx *expression patterns is not dissociated between the different dentitions within a given organism [18].

We show here that the first developing teeth and caudal primary scales form through four common typical stages that correspond to the common stages we previously identified for oral and pharyngeal odontogenesis in mouse and teleosts [12,17]. We have identified six *Dlx *genes in the dogfish and analysed their expression patterns in teeth and caudal primary scales at the histological level. Three of them showed strong transcription in both structures (*Dlx3*, *Dlx4 *and *Dlx5*) whereas lower transcription levels could be detected for *Dlx1 *in dermal denticles and teeth, and for *Dlx2 *in dermal denticles. We observed very little difference in the transcription patterns of a given *Dlx *gene between teeth and caudal primary scales, except for the lack of *Dlx2 *transcription specifically in tooth buds. These results strongly suggest that a single set of *Dlx *genes is involved in oral tooth and dermal denticle development in the dogfish and therefore strongly support the hypothesis of serial homology between these odontodes. In this context, we propose that *Dlx *genes belong to a core set of developmental genes involved in all odontode development in gnathostomes. Our results imply that the GRN involved in odontode formation could have been initiated at several location (skin, mouth, oral cavity, pharynx) by simple heterotopy during the course of evolution and therefore represent the first detailed molecular support for the "inside and out" model.

## Results

### Tooth and dermal denticle development

**Figure 1:**
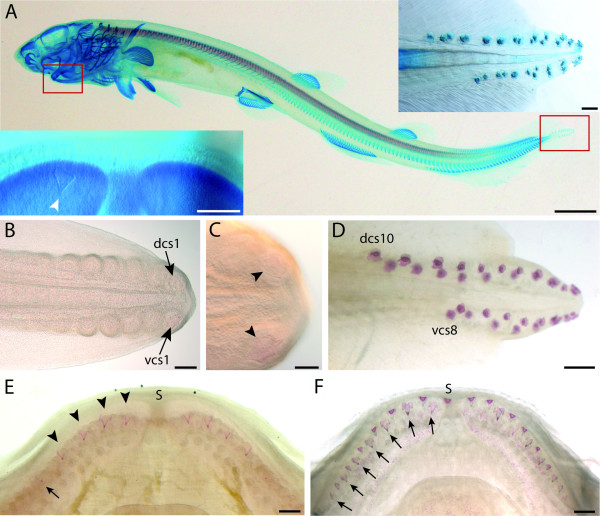
**Localization and embryonic development of caudal primary scales and oral teeth in the dogfish**. A: ventro-lateral view (anterior is to the left) of a 6 cm long embryo stained with alcian blue (cartilage) and alizarin red (prismatic calcified cartilage and dentine); insets: left panel is a ventral view (anterior is to the top) of the lower jaw showing the first mineralized tooth (white arrowhead), right panel is a close-up lateral view (anterior is to the left) of the very tip of the tail showing the bilateral dorsal and ventral rows of caudal primary scales. B-D: caudal primary scale organisation, anterior is to the left and dorsal to the top in all pictures. B: left lateral view of the tail of a 3 cm long embryo after alizarin red staining showing seven buds in the dorsal row and six on the ventral one. Rostral buds are less developed than caudal ones; only the caudal-most buds show alizarin red staining (close up in C, arrowhead). D: left lateral view of the tail of a 7 cm long embryo showing alizarin red stained caudal primary scales. All primary scales are fully developed. Primary scales of the right side are out of focus. E-F: dorsal views of lower jaws after alizarin red staining, anterior is to the top; E: 7 cm long embryo, the four first teeth of the first row are stained (arrowheads) and the following are beginning to mineralize (arrow); F: 7.5 cm long, teeth of the second row (arrows) intercalate between teeth of the first row. Scale bars: A: 0.4 cm; insets 400 μm; B: 200 μm; C: 100 μm; D-F: 400 μm; dcs: dorsal caudal scale, vcs: ventral caudal scales, s: symphysis.

As mentioned by Mellinger and Wrisez [22] the first developing dermal denticles (caudal primary scales) and teeth could be observed at the very tip of the tail and the lower jaw, respectively (Figure 1A, insets). In order to follow the pattern of mineralization in the first developing teeth and dermal denticles, jaws and tails dissected from dogfish embryos measuring from 2 cm to 9 cm were stained with alizarin red (Figure 1B-F). At the tip of the tail, buds of caudal primary scales were first observable in 2.5 cm long embryos. As previously described [22], caudal scale development progressed in a posterior to anterior wave in the terminal part of the tail: 3 cm-long embryos showed alizarin red staining only in the very caudal scales while very young scale buds were still at early stage of development in the anterior part of the primary scale-forming area (Figure 1B-C). In 3.4 cm long embryo, four scales were mineralized in the dorsal and ventral parts of the tail (not shown). In 4 cm long (not shown) and older embryos, the full set of caudal primary scales was stained with alizarin red (Figure 1A, D,) although the most rostral scales were not erupted yet (not shown). We chose to name these scales Dorsal or Ventral Caudal Scales (dcs or vcs) respectively -1 to -10 and -1 to -8, from caudal to rostral: number refers to the rank of apparition during development, but few variations in scale number were observed between specimens.

The detailed pattern of tooth development was difficult to identify by alizarin red staining: the first mineralized tooth could be detected in embryos reaching 6 cm long while there were at least five on each quadrant in 7 cm long embryos (Figure 1A, E). Despite some individual variations, the first tooth bud generally appeared lateral to the symphysis, and then two other tooth buds developed on both sides of the first tooth. Additional teeth subsequently developed on the jaw margin, from the symphyseal (distal) portion towards the articulation (proximal). New tooth buds also developed between teeth of the first row, in a more posterior second row of teeth, starting in 7.5 cm long embryo (Figure 1F). A third row of tooth buds was observable in 8.5 cm long embryos (not shown).

### Histological characterization of tooth and dermal denticle developmental stages

**Figure 2:**
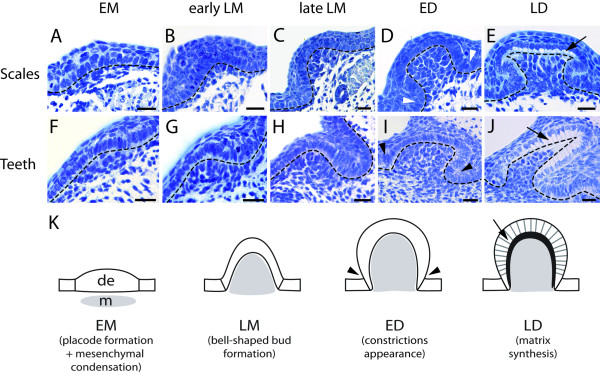
**Histological definition of four developmental stages for caudal primary scale and tooth development**. Transversal sections of a tail from a 3 cm long embryo (A-E) and of lower jaws from 4 cm (F-H) and 5.5 cm (I-J) long embryos coloured with *Nissl *stain (cresyl violet - *thionin*), scale bar: 25 μm. K: diagram summarising the four common stages for odontode development in the dogfish. EM, early morphogenesis- thickening of the dental epithelium (d.e.), condensation of the mesenchyme (m, coloured grey); LM, late morphogenesis- bell shape bud formation; ED, early differentiation- a constriction appears at the bottom of the bud (arrowheads); LD, late differentiation- matrix secretion (arrow).

In order to depict the modifications of the mesenchymal and epithelial compartments through tooth and dermal denticle development, we performed histological sections and *Nissl *stain (cresyl violet - *thionin*) coloration on dissected tails of 3 cm long embryos and on lower jaws for embryos ranging from 4 cm to 5.5 cm long (Figure 2).

The first developing oral tooth and caudal primary scale buds showed the same successive cellular and histological stages that those previously shown to be common between oral and pharyngeal tooth development in osteichthyans [12,17]. In addition, cellular and histological aspects of scale bud development were similar to tooth bud stages. In both structures, the early morphogenesis stage (EM) started with the placode formation: the shape of odontogenic epithelial cells changed from cubic to prismatic and the underlying mesenchyme began to condensate (Figure 2A, F). At late morphogenesis stage (LM), the epithelium progressively folded and enclosed the mesenchymal compartment resulting in a bell-shaped bud. At that stage, we could distinguish between early LM stage when the epithelium started to fold (Figure 2B, G) and late LM stage when the epithelium fold was more pronounced and the bud exhibited a typical bell shape (Figure 2C, H). The third stage, ED (early differentiation), was characterized by a constriction that could be observed at the basis of the bell on both scale and tooth buds (Figure 2D, I). During the last stage, LD (late differentiation), epithelial cells had their nucleus shifted towards the apical pole and showed secreting vesicles in their basal cytoplasm. The first signs of matrix deposition confirmed that ameloblasts were fully differentiated (Figure 2E, J). Differentiation of the odontoblasts was not included in this analysis as no histological sign could be identified showing their synthesis activity. These observations are summarised in the diagram in Figure 2K.

### Overall expression of *Dlx *genes in first forming teeth and dermal denticles

Two segments for each *Dlx *coding sequences were amplified from the dogfish genome by degenerate PCR based on the six *Dlx *sequences identified in *Triakis semifasciata *[29]. These segments were concatenated and phylogenetic analyses were performed to check their orthology to gnathostome *Dlx1 *to *Dlx6 *(see Additional file 1). The amplified sequences were used to synthesize anti-sense RNA probes against the *Dlx *mRNAs in the dogfish (Additional file 2).

**Figure 3:**
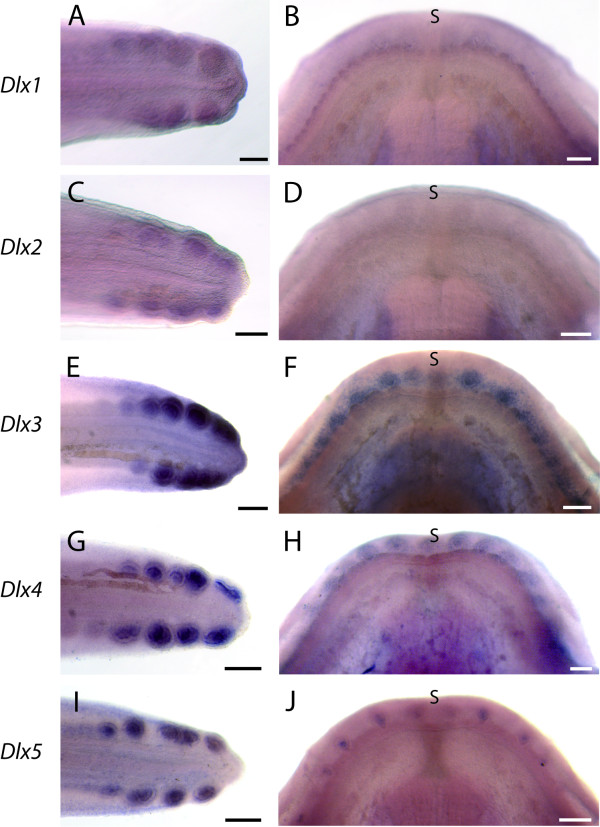
**Whole mount views of *Dlx *gene expression during caudal primary scale and tooth development**. *In situ *hybridizations against *Dlx1 *(A-B), *Dlx2 *(C-D), *Dlx3 *(E-F), *Dlx4 *(G-H) and *Dlx5 *(I-J) mRNAs. Left lateral view of tails from embryos at stage 30 (A, C, E, G and I), anterior to the left, dorsal to the top. Four dorsal scale buds and three ventral buds are stained in A, five dorsal and four ventral scale buds in C, E, G and I. Dorsal views of lower jaws (B, D, F, H and J), anterior to the top, from 4.9 cm (B and D), 4.5 cm (F), 4.6 cm (H) and 4.7 cm (J) long embryos. Scale bars: 200 μm, s: symphysis.

We performed *in situ *hybridizations on dissected tails of 2.5 cm to 3 cm long embryos (stage 29-31) and on lower jaws for embryos ranging from 4 cm to 5.5 cm long, in order to describe gene expression in the first developing teeth and caudal primary scales through their whole developing process. Transcripts were detected at high levels in both structures for three out of six *Dlx *genes: *Dlx3*, *Dlx4 *and *Dlx5 *(Figure 3E-J). Lower levels of expression could be detected with the *Dlx1 *and *Dlx2 *probes in developing caudal primary scales, and with *Dlx1 *probes in developing teeth (Figure 3A-D). *Dlx6 *transcripts were never detected, either in tooth or scale buds (not shown).

### Tissue specific expression of *Dlx *genes during dermal denticle development

In order to obtain a detailed description of the *Dlx *gene expression patterns during the development of dermal denticles in the dogfish, we prepared histological sections of the whole-mount hybridized tails, and assigned each caudal primary scale bud to one of the four stages of development described in Figure 2. Caudal primary scale development lasts over weeks and expression patterns may change along a given developmental stage. We observed as many buds as possible for each stage, and separated the LM into an early and a late LM, in order to obtain a more detailed view of the dynamic changes of expression patterns. For a given probe, the expression dynamic is identical in at least the sixteen first caudal primary scales (four first dorsal and ventral caudal scales on each lateral side), so the values given for each stage in the following paragraph result from pooling together our observations as summarized in Table 1.

**Table 1:** Occurrence of positive staining in the epithelial and mesenchymal compartments of developing caudal primary scale buds after *in situ *hybridization

Epithelium						Mesenchyme			
		
	EM	LM	ED	LD	EM	early LM	late LM	ED	LD
*Dlx1*	0/4	5/16	19/34	29/34	0/4	0/7	0/9	11/34	0/34
*Dlx2*	11/13	31/36	16/28	9/11	0/13	6/17	17/19	27/28	0/11
*Dlx3*	25/25	28/28	26/26	28/28	0/25	7/19	9/9	20/26	0/28
*Dlx4*	20/20	65/65	36/36	22/22	0/20	5/35	29/30	31/36	6/22
*Dlx5*	18/18	53/53	35/35	26/26	0/18	0/27	20/26	35/35	13/26

Data are calculated as the number of buds with positive staining out of the number of observed buds (see legend of Figure 2 for stage name abbreviations)

**Figure 4:**
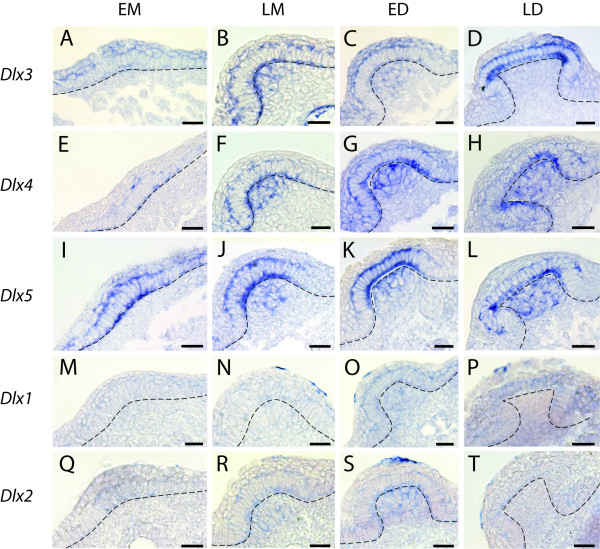
**Histological analysis of *Dlx *gene expression patterns during caudal primary scale bud development**. Transversal sections of whole mount hybridized tails from embryos at stages 29 to 31 illustrating the expression patterns of *Dlx3 *(A-D), *Dlx4 *(E-H), *Dlx5 *(I-L), *Dlx1 *(M-P) and *Dlx2 *(Q-T) during the development of the scales. Embryos are at stages 29 (B, E, F, I, M, N and Q), 30 (A, C, G, H, L, O, R-T) and 31 (D, K and P). EM, LM, ED and LD legends are as in Figure 2. For the LM stage, we illustrate the late LM stage when *Dlx *genes are predominantly expressed in the mesenchyme of scale buds. Dotted lines indicate the basal lamina separating the dental epithelium and the underneath mesenchyme. Scale bars: 25 μm.

During caudal primary scale development, we detected low levels of *Dlx3 *transcripts in the thickened epithelium of all bud placodes (EM, n = 25/25, Figure 4A). *Dlx3 *transcripts were then detected at higher levels in the epithelial compartment of both the LM stage and ED stage (n = 28/28 and n = 26/26, respectively, Figure 4B, C). Finally, transcription of *Dlx3 *was detected only in the epithelial layer in the beginning of the LD stage (n = 28/28, Figure 4D). *Dlx3 *transcripts were detected in the mesenchyme in only some of the scale buds during the LM stage (n = 16/28): early LM group showed both negative and positive buds (n = 7 positive out of 19 early LM) while positive staining was always observed in late LM buds (n = 9/9, Figure 4B). Transcription of *Dlx3 *was therefore progressively initiated during the beginning of the LM stage. *Dlx3 *transcripts were apparent in the mesenchyme of ED scale buds (n = 20/26) but undetectable in LD stages, showing a negative regulation of *Dlx3 *during the LD stage (Figure 4C-D).

Transcripts of *Dlx4 *were detected with the same expression pattern: in the epithelium of caudal primary scale buds during the EM (n = 20/20, Figure 4E), LM (n = 65/65, Figure 4F), ED (n = 36/36, Figure 4G) and LD (n = 22/22, Figure 4H) stages and in the mesenchyme of scale buds at the LM (34/65), ED (31/36) and LD (n = 6/22). Again, during the LM stage, positive staining was mainly observed in more late LM buds (n = 29 positive out of 30 late LM stage, Figure 4F) while positive buds were rarely observed in the early LM group (n = 5 positive out of 35 early LM). Positive staining in the mesenchyme of LD stages was more frequently observed in less developed buds, suggesting that *Dlx4 *expression is turned off early during LD.

We also detected *Dlx5 *transcripts in the epithelium of caudal primary scale bud during the four stages of development (EM, n = 18/18; LM, n = 53/53; ED n = 35/35; LD, n = 26/26), and in the mesenchymal compartment of the LM (n = 20/53 positive, of which n = 20 in the late LM stages out of 26 late LM buds), ED (n = 35/35) and LD (n = 13/26) stages (Figure 4I-L).

**Figure 5:**
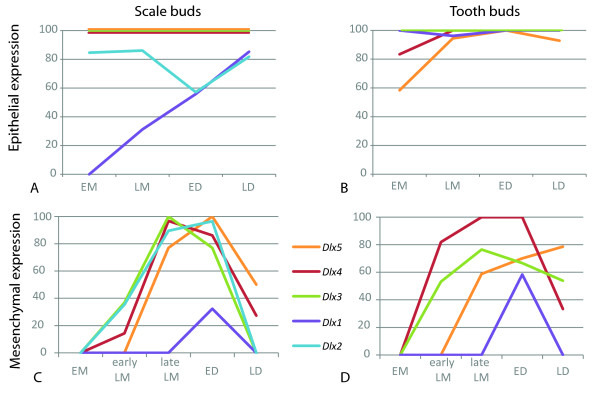
**Dynamics of *Dlx *gene expression in the epithelial and mesenchymal compartments of developing caudal primary scales and teeth**. Graphical representation of data presented in Tables 1 and 2. For each gene and each developmental stage, the percentage of positive buds over the total number of observed buds is plotted and a curve connecting these plots is drawn. Epithelial (A, B) and mesenchymal (C, D) compartments in caudal primary scales (A, C) or teeth (B, D). See legend of Figure 2 for abbreviations of the developmental stages.

Histological sections on hybridized tails revealed no detectable expression of *Dlx6 *at any stage (not shown), while *Dlx1 *and *Dlx2 *transcripts were detected at low intensity (Figure 4M-T). We used these same *Dlx *probes on whole-mount embryos at early stage of organogenesis (see Additional file 3), and could get strong signals for every *Dlx *genes here identified (note that probe lengths are equivalent for all six genes). We concluded that our results with *Dlx1 *and *Dlx2 *probes were not due to experiment or probe artefact, but most likely were a consequence of low expression levels for these two genes during caudal primary scale development. *Dlx1 *transcripts were detected in the epithelium of scale buds at the LM (n = 5/16, all of them from the late LM group, 10 late LM buds), ED (n = 19/34) and LD (n = 29/34) stages as well as in the mesenchyme of ED (n = 11/34) scale buds (Figure 4M-P), showing a progressive up-regulation of *Dlx1 *in the late LM/early ED stage. *Dlx2 *transcripts were detected in the epithelium of caudal primary scale buds at the EM (n = 11/13), LM (n = 31/36), ED (n = 16/28) and LD (n = 9/11) stages. *Dlx2 *expression was also detected in the mesenchymal compartment of scale buds during LM (n = 23/36) stage, most of the positive buds being in the late LM group (n = 17 out of 19 late LM buds). Transcripts were then detected during ED (n = 27/28) stage but undetectable in LD (Figure 4Q-T), showing activation of *Dlx2 *transcription during the LM stage and down-regulation early in the LD stage. The dynamics of each *Dlx *gene expression in the epithelial and mesenchymal compartments of primary scale buds are graphically illustrated in Figure 5A and 5C.

### Tissue specific expression of *Dlx *genes during tooth development

**Figure 6:**
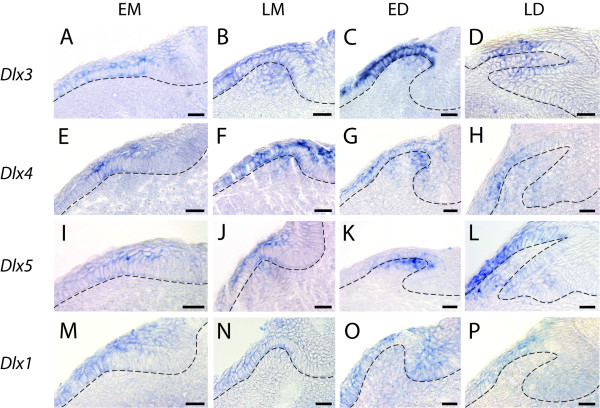
**Histological analysis of *Dlx *gene expression patterns during tooth development**. Transversal sections of hybridized lower jaws of 4 cm to 5.5 cm long embryos illustrating the expression patterns of *Dlx3 *(A-D), *Dlx4 *(E-H), *Dlx5 *(I-L) and *Dlx1 *(M-P) during the development of oral teeth. Lower jaws of 4 cm long embryos (A, I and M), 4.3 cm (E), 4.5 cm (B, F, and J), 4.6 cm (G, N and O), 5 cm (C, D and K), 5.1 cm (P), 5.4 cm (H) and 5.5 cm (L). We illustrate only the late LM stage when *Dlx *genes are predominantly expressed in the mesenchyme of tooth buds. Legends are as in Figure 2 and 4. Scale bar: 25 μm.

As described for dermal denticles, we pooled our observations for the eight first teeth (four first teeth on each hemi-jaw) and summarized our results in Table 2. During tooth development, *Dlx3*, *Dlx4 *and *Dlx5 *transcription started in the epithelial compartment of EM buds and was maintained in LM, ED and LD (Figure 6A-L and Figure 5B). Expression in the mesenchymal compartment started during the LM stage (*Dlx3*, n = 17/31; *Dlx4*, n = 14/22; *Dlx5*, n = 10/37) and most of the positive buds were found in the late LM group (*Dlx3*, n = 13/17, Figure 6B; *Dlx4*, n = 10/10, Figure 6F; *Dlx5*, n = 10/17, Figure 6J and 5D). Expression in the mesenchymal compartment was maximum for *Dlx3 *and *Dlx4 *during the ED stage (n = 12/18 and n = 13/13, respectively, Figure 6C, G and 5D) but down regulation of their expression was apparent during the LD stage (positive tooth buds: n = 7/13 for *Dlx3*, Figure 6D, and n = 7/21 for *Dlx4*, Figure 6H and 5D). *Dlx5 *transcripts were detected in the mesenchyme of some of the tooth buds at the ED stage (n = 14/20, Figure 6K) and was maximal in LD stage buds (n = 11/14, Figure 6L and 5D). Expression of *Dlx2 *and *Dlx6 *could not be significantly detected by *in situ *hybridization followed by histological sections. Among 42 *Dlx2 *hybridized teeth, we were able to detect a very weak signal in the epithelium during the LM stage of six developing tooth buds. We decided to consider that these data were not significant, but we cannot exclude that the level of *Dlx2 *transcription was too low to be detected by the *in situ *hybridization technique. On the contrary, *Dlx1*consistently showed low levels of expression in developing tooth buds: transcripts were detected in the epithelium of all four stages of development (EM, n = 5/5, Figure 6M; LM, n = 25/26, Figure 6N; ED, n = 12/12, Figure 6O; LD, n = 6/6, Figure 6P; see also Figure 5B), and transient expression in the mesenchyme of ED buds could be observed (n = 7/12, Figure 6O and 5D).

**Table 2:** Occurrence of positive staining in the epithelial and mesenchymal compartments of developing tooth buds after *in situ *hybridization

	Epithelium					Mesenchyme			
		
	EM	LM	ED	LD	EM	early LM	late LM	ED	LD
*Dlx1*	5/5	25/26	12/12	6/6	0/5	0/13	0/13	7/12	0/6
*Dlx3*	9/9	31/31	18/18	13/13	0/9	4/14	13/17	12/18	7/13
*Dlx4*	5/6	22/22	13/13	21/21	0/6	4/12	10/10	13/13	7/21
*Dlx5*	7/12	34/37	20/20	13/14	0/12	0/20	10/17	14/20	11/14

Data are calculated as the number of buds with positive staining out of the number of observed buds (see legend of Figure 2 for stage name abbreviations)

## Discussion

### Dermal denticle and tooth development progress through common developmental stages

It has long been considered that oral teeth and dermal denticles display developmental similarities [1-3]. The histological data we obtained for embryonic oral tooth and caudal primary scale development in the dogfish are in accordance with those obtained previously in other elasmobranch species or at later stages of dogfish development [20,21]. In addition, our morphological and histological observations show that teeth and scales share morphogenetic similarities through four developmental stages, which are also shared with mammalian oral teeth and teleost pharyngeal or oral teeth [12,17,30]. As a consequence, we postulate here that all odontodes, whatever their location, develop through four common successive stages including the formation of a placode (EM), shaping of the bud (LM), differentiation of ameloblasts and odontoblasts (ED) (as defined in osteichthyans and as deduced from the observation of fully functional ameloblasts in LD buds in the dogfish), and matrix deposition (LD). Similar organization and composition of the fully developed teeth and dermal denticles, as well as similar stages of development, support the hypothesis of serial homology between epithelial mineralized structures.

### Serial homology of odontodes is molecularly supported by non-dissociation of *Dlx *expression patterns in caudal primary scales and teeth

To further explore the potential link of serial homology between odontodes we analysed the expression pattern of six *Dlx *genes in *Scyliorhinus canicula*. We assumed that these six genes constitute the whole set of *Dlx *genes in the dogfish, which also was the probable ancestral state in gnathostomes [29], even if we cannot exclude that individual duplications occurred in the dogfish lineage. No expression of *Dlx6 *could be detected in either tooth or scale buds. *Dlx3*, *Dlx4 *and *Dlx5 *were expressed in the epithelial compartment during all four common stages of caudal primary scale and tooth development (Figure 5). In the mesenchymal compartment, transcription of *Dlx3*, *Dlx4 *and *Dlx5 *was initiated during the LM stage and then down-regulated shortly after the ED stage for *Dlx3 *and *Dlx4 *while expression of *Dlx5 *was still on during the LD stage. The expression dynamics of each of these three genes show only subtle differences between caudal primary scale and tooth. First, more than 40% of the tooth buds we examined show no transcription of *Dlx5 *in the epithelium during the initiation of expression at EM stage whereas it is expressed in the epithelium of all caudal primary scale buds (Figure 5A and 5B). Similarly, *Dlx5 *transcription in the mesenchyme is never detected in 100% of tooth buds as it is in scale buds (Figure 5C and 5D). Second, the dynamic of transcription of *Dlx3 *is identical in the epithelium of both tooth and caudal primary scale (Figure 5A and 5B) but the expression in the mesenchyme at ED is detectable in all scale buds stage but only in about 80% of the tooth buds (Figure 5C and 5D). Expression of *Dlx1 *was also similar in the mesenchymal compartment of both caudal primary scale and tooth buds, with transient expression during the ED stage. However, the *Dlx1 *expression pattern showed heterochrony in the epithelial compartment as transcription started during the LM stage in scale buds, while it was already active in the EM stage in tooth buds. Note that *Dlx1 *signal was very weak compared to *Dlx3*, *Dlx4 *and *Dlx5 *and we cannot exclude that *Dlx1 *expression was too weak to be detected during the early morphogenesis stages. The main difference observed in this study is the complete lack of expression of *Dlx2 *during tooth development, while the expression dynamic of this gene was similar (although weaker) to those of *Dlx3 *and *Dlx4 *during scale bud development. In conclusion, we show that the tissue specific expression patterns of each *Dlx *genes are nearly identical (except for *Dlx2*) during oral tooth and dermal denticle development in the dogfish. Taken together with our previous data on oral and pharyngeal dentitions, the results presented here show that the *Dlx *genes expression patterns did not undergo dissociation between odontodes (teeth, denticles, scales) that form at different locations (mouth, pharynx, skin) in a given species. Therefore, all odontodes of a given organism appear to be serially homologous because they bud through the redeployment of common developmental stages associated with a similarly regulated set of *Dlx *genes.

### Different *Dlx *expression patterns during odontode development among jawed vertebrates

**Figure 7:**
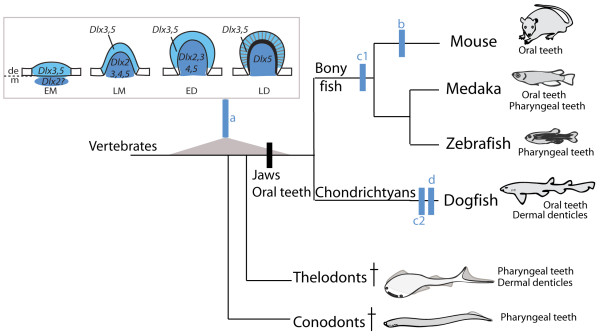
**Theoretical tree of the relationships between extant gnathostomes and fossil species**. The evolutionary scenario described in the discussion is illustrated, including the diversification of vertebrate odontodes and hypotheses for the modification of *Dlx *gene expression patterns over time: a- proposed ancestral situation (note that the early *Dlx2 *mesenchymal expression depends on the alternative hypotheses in c); b- loss of epithelial *Dlx3 *and *Dlx5 *expression; c- two equi-parsimonious hypotheses: c1 = gain in bony fish/c2 = loss in chondrichthyans of the *Dlx2 *early mesenchymal expression; d- loss of *Dlx2 *expression in teeth but not in dermal denticles.

In order to gain insights into the evolution of *Dlx *expression patterns during gnathostome odontode development we compared our results with those published in mouse [26], zebrafish [12] and medaka [18]. A summary of expression patterns published for the mouse *Mus musculus *(m), zebrafish *Danio rerio *(d), medaka *Oryzias latipes *(o) and dogfish *Scyliorhinus canicula *(s) are presented in Table 3. Note that the results recently obtained for *Dlx *expression during pharyngeal tooth development in an African cichlid (*Astatotilapia burtoni*) [28] were consistent with what had previously described for *Dlx *expression in the medaka and were therefore omitted in the Table 3 in order to clarify the analysis. Contrasting with the non dissociation of *Dlx *expression patterns in odontodes within a given species (this work and [18]), the set of genes expressed varies highly between species: *Dlx1 *is never expressed during zebrafish or medaka odontogenesis while *Dlx6 *is not expressed during dogfish or zebrafish odontogenesis. This observation suggests that a specific combination of *Dlx *genes was not strictly constrained during gnathostome evolution, probably because the redundancy between the different paralogs favoured function shuffling. On the other hand, *Dlx2*, *3*, *4*, and *5 *are transcribed in all three species even though their expression patterns vary, especially in the epithelial compartment. Common points are restricted to: (i) early epithelial transcription for *Dlx3 *(EM); (ii) mesenchymal transcription for all four genes during LM and ED (note two exceptions, no expression of *dlx4b *in the mesenchyme of LM buds in medaka, no expression of *dlx3b *in the mesenchyme of ED buds in zebrafish); (iii) late (LD) mesenchymal transcription for *Dlx5*. Given the homology relationship proposed between gnathostome odontodes, these similar features might be inherited from the gnathostome last common ancestor (see the ancestral expression pattern, hypothesis a, in Figure 7). Another trend is highlighted with this comparison: expression of *Dlx3 *and *Dlx5 *in the epithelium at all stages is observed in all species but the mouse (except for *Dlx3 *expression in EM stage).

**Table 3:** *Dlx *gene expression patterns during odontode development in gnathostomes

	EM		LM		ED		LD	
				
	E	M	E	M	E	M	E	M
*Dlx1*	(s)	-	s	m	s	ms	s	m
*Dlx2*	md(s)	mod	d(s)	**mod(s)**	d(s)	**mod(s)**	md(s)	d
*Dlx3*	**mods**	o	*ods*	**mods**	*ods*	mos	*ods*	mo
*Dlx4*	ds	d	ds	mds	ds	**mods**	ds	mo
*Dlx5*	*(o)ds*	o	*(o)ds*	**mods**	*ods*	**mods**	*ods*	**mods**
*Dlx6*	-	-	-	m	-	mo	-	mo

Data were showed for the six paralogous groups, as published in mouse [26], zebrafish [12], medaka [18] and dogfish (this study) (expression patterns of co-orthologs such as zebrafish *dlx2a *and *dlx2b *were added up). Species initials are indicated when positive expression has been published in the epithelial (E) or mesenchymal (M) compartments in the different stages. See legend of Figure 2 for abbreviations of developmental stages; m: mouse, *Mus musculus*; o: medaka, *Oryzias latipes*; d: zebrafish, *Danio rerio*; s: dogfish, *Scyliorhinus canicula*; -: no expression detected. Expression of dogfish *Dlx2 *that was observed only in scale buds is indicated in brackets, expression of medaka *dlx5a *observed only in oral teeth is indicated in brackets. Bold shows characteristics conserved among all gnathostomes, italics indicate characteristics possibly lost in mouse.

This may be interpreted as a specific loss in the lineage leading to the mouse (sarcopterygian), and could even be viewed as a single evolutionary event if one considers that the epithelial expression of *Dlx3 *and *Dlx5 *is up-regulated by one single activator. If considered as a single evolutionary event, the most parsimonious scenario to explain these data would be that *Dlx3 *and *Dlx5 *were transcribed in the epithelium of odontode buds at all stages in the gnathostome last common ancestor, and that this trait was lost secondarily in the sarcopterygian lineage leading to the mouse (see hypothesis b, in Figure 7). This result could be correlated to the different roles taken by the epithelial compartment in sarcopterygians as opposed to non-sarcopterygians [31]. In non-sarcopterygians (chondrichthyans and actinopterygians), the outer-most layer of mineralized tissue (enameloid) is synthesized by a cooperation between the mesenchymal (neural-crest derived odontoblasts) and the epithelial (ameloblasts) compartments [32]. On the contrary, the sarcopterygian outer mineralized layer (best described in amniotes) is composed of enamel, which has been showed to be synthesized exclusively from the epithelial layer of ameloblasts with specific secretory characteristics [33].

Another prominent result is the lack of early *Dlx2 *expression (during EM stage) in the mesenchyme of tooth and dermal denticle buds in the dogfish. This early expression in the mesenchymal compartment was observed in the mouse, zebrafish and medaka, and is a specificity of the *Dlx2 *orthologs. Two equi-parsimonious scenarios can be proposed: (i) the early mesenchymal expression of *Dlx2 *is a novelty acquired in the last common ancestor of bony fish only; (ii) the early mesenchymal expression of *Dlx2 *is an ancestral gnathostome characteristic, and it has been lost in the chondrichthyan lineage (hypotheses c1 and c2 in Figure 7). Note that the lack of *Dlx2 *expression in dogfish teeth is most probably a derived state (hypothesis d in Figure 7) as *Dlx2 *expression is detected in dogfish scales. Other data presented in Table 3 do not allow to propose one most parsimonious scenario for expression pattern evolution: one-species characteristic i.e. apomorphy, or two-species characteristic leading to convergence (mouse (m) + medaka (o), or zebrafish (d) + dogfish (s)).

## Conclusion

Here, we present a detailed comparison of tooth and dermal denticle development in the dogfish that show that odontodes should accurately be considered as serial homologous structures. Indeed in a given species, the development of the different odontodes involves (i) the localised redeployment of the same developmental stages, and (ii) the localised redeployment of the same *Dlx *expression patterns (but *Dlx2*). We reasoned that, if an ancestral regulation of *Dlx *expression had dissociated and evolved independently in the different odontodes after its cooption at different locations, there should be more differences between expression patterns when comparing structures in one organism (serial homology) than when comparing homologous structures between extant gnathostomes (specific homology). However, we observe more divergence between the *Dlx *expression patterns of different species than between serial homologous structures within a species such as dogfish teeth and dermal denticles (see Table 3) or teleost oral and pharyngeal teeth, as shown in the Additional file 4. We conclude that the reiterated expression of the dynamic *Dlx *patterns in teeth and dermal denticles within a given organism reveals that the different odontodes form from the reiterated expression of a single GRN. Our results therefore provide the first detailed molecular supports for the "inside and out" model [19] that propose that all odontodes are serially homologous structures that form through the control of a common odontode gene regulatory network (oGRN). This ancestral oGRN may include, in addition to the *Dlx *family, several other genes such as *Shh*, *Epha4*, and *Runx*, that were shown to be expressed during both scale and tooth development in chondrichthyans [23-25], and during oral and/or pharyngeal tooth development in mouse and/or teleost fish [34,35]. The evolutionary scenario for the relative time of appearance of the different odontodes depends on the phyletic relationships between fossil agnathans such as conodonts and thelodonts, extant agnathans, and extant gnathostomes, which remain controversial [7,8,36]. Our results support the hypothesis that the GRN involved in the development of serially homologous odontodes has not dissociated over gnathostome evolution. This implies that, during the course of evolution, the diversity of gnathostome odontodes arose from independent gains of expression territories of a single ancestral GRN by simple heterotopy. Therefore, molecular developmental data would not allow comparing odontodes in order to evaluate their divergence over evolutionary time. As a consequence, only additional paleontological data can shed a new light on the evolutionary events leading to the diversity of odontodes.

## Methods

### Animals

Dogfish (*Scyliorhinus canicula*) embryos were obtained from the Station de biologie marine (Roscoff, France, CNRS and MNHN). All embryos were maintained at 17°C in sea water until they reach the correct developmental stage, defined by their total length. They were dissected and then fixed 48 hours at 4°C in a phosphate buffered saline solution containing 4% paraformaldehyde (PFA). Embryos were then dehydrated in methanol and stored at -20°C.

### Alizarin red staining

Whole-mount embryos stored in methanol were progressively transferred in 0.5% KOH solution. They were then coloured over-night in an alizarin red solution (0.001%) in 0.5% KOH at room temperature. After their progressive transfer to glycerol, they were photographed under bright field.

### Double alcian blue and alizarin red staining

Whole-mount embryos (more than 5 cm long) were fixed in buffered 10% formaldehyde for a day, then rinsed in distilled water and transferred to 70% ethanol for storage. They were stained in filtered alcian blue solution (alcian blue 200 mg/L in 70% ethanol-30% glacial acetic acid) for 48 hours, rinsed in decreasing ethanol bathes, to distilled water, and then in a 30% saturated sodium borate solution. They were then digested in a trypsin solution (1% in 30% saturated sodium borate solution, renewed when blue) until the specimen is cleared. Specimens were then transferred in a 0.5% KOH solution to be stained with alizarin red.

### Cloning *Dlx *coding sequences

Amplification of the first and third exon of each *Dlx *coding sequence was made with degenerated primers designed on the published sequences of *Triakis semifasciata Dlx *genes [29]. Dlx probe positions are shown on Additional file 2, and primer sequences are given in Additional file 5. PCR products were cloned in pGEM-T Easy vector (Promega). Antisense RNA digoxigenin-UTP probes were prepared using SP6 or T7 polymerase, according to the orientation of the insert in the plasmid.

### Histological sectioning

Dissected jaws and tails from embryos stored in methanol at -20°C were put through several baths of absolute ethanol, then in butanol and finally embedded in paraplast for 10 μm cross-sections. Sections were then coloured *with Nissl *stain (cresyl violet - *thionin*).

### Whole mount *in situ *hybridizations

Whole mount *in situ *hybridizations were performed according to standard protocol [24] using two antisens RNA probes for each assay on dissected jaws (from 4 to 5.5 cm embryos long) or tails (from 2.5 to 3 cm embryos long). Proteinase K treatments (10 μg/mL) were adapted for dissected jaws and tails: 30 min at room temperature for tails, twice 30 min for jaws. The colour detection step was performed using the NBT-BCIP reaction. We assayed this protocol on at least four embryos for each developmental stage and with positive control (earlier embryo with known restricted expression pattern with the same probe, see Additional file 3).

### Expression pattern analysis

The dissected jaws and tails were post-fixed in 4% PFA after whole mount *in situ *hybridization, then cleared and stored in glycerol at 4°C to be photographed under bright field. Whole-mount hybridized dissections were put through several baths of absolute ethanol, then in butanol and finally embedded in paraplast for 10 μm cross-sections. Negative whole-mount detections were also verified after histological sections.

## Authors' contributions

MDT, VBB, SO and FB produced and analysed the morphological and *in situ *hybridization data. DC carried out the gene characterization and phylogenetic analyses. MDT, VBB and PL drafted the manuscript. VBB and DC designed the study. All authors have read and approved the manuscript.

## Supplementary Material

Additional file 1 **Assignment of the *Dlx *probes to their group of orthologues**. In order to assign each *Dlx *probe to an orthologous group, phylogenetic analyses were performed. The *Dlx *gene sequences of *Triakis semifiasciata *(leopard shark), *Homo sapiens *(human), *Gallus gallus *(chicken), *Xenopus tropicalis *(xenopus), *Danio rerio *(zebrafish) were retrieved from Ensembl release 49 and GenBank release 164. Multiple *Dlx *amino acid sequences were aligned using ClustalX (Thompson et al. 1997) with manual optimization using MUST software (Philippe 1993). Regions of ambiguous homology were removed. Evolutionary distances were computed using the Poisson correction and a neighbor-joining tree was obtained using MEGA4 (Tamura et al. 2007). The robustness of the tree nodes was estimated by a bootstrap test (1000 replicates). Philippe H. 1993. MUST, a computer package of Management Utilities for Sequences and Trees. Nucleic Acids Res 21:5264-5272. Tamura K, Dudley J, Nei M, and Kumar S. 2007. MEGA4: Molecular Evolutionary Genetics Analysis (MEGA) software version 4.0. Mol Biol Evol 24:1596-1599. Thompson JD, Gibson TJ, Plewniak F, Jeanmougin F, and Higgins DG. 1997. The CLUSTAL_X windows interface: flexible strategies for multiple sequence alignment aided by quality analysis tools. Nucleic Acids Res 25:4876-4882. Click here for file

Additional file 2 **Position of *Dlx *probes on the coding sequences of *T. semifasciata***. The position of a probe is indicated by a grey line. The position of introns (I), the position of homeobox sequences (black box) and the length of each coding sequence (cds) and of each probe are indicated. Click here for file

Additional file 3 **Test of *Dlx *probes activity during early stages of embryogenesis in the dogfish**. Lateral view of whole mount *in situ *hybridized embryos with the *Dlx *probes designed for the 6 dogfish *Dlx *genes. For each panel, the name of the probe is indicated up right and the stage of the hybridized embryo down right. Click here for file

Additional file 4 **Statistical evaluation of differences between *Dlx *expression patterns in gnathostome odontode development**. A. Matrix describing the expression pattern of each *Dlx *gene from the mouse, zebrafish, medaka and dogfish, during all four odontode developmental stages (see text for a description), either in the epithelial (e) or mesenchymal (m) compartment. Data are as of Table 3, but different columns were made for oral (o) versus pharyngeal (p) teeth in medaka, or scales (s) versus oral teeth (o) in dogfish. B. Neighbor Joining tree inferred with a pairwise distance (number of differences) matrix estimated with the matrix of characters shown in A. Click here for file

Additional file 5 **Primer sequences**. Primer names follow the description in the Additional file 2. Click here for file
