# Supplementary figures and images for: The homology of odontodes in gnathostomes: insights from Dlx gene expression in the dogfish, Scyliorhinus canicula
Source: BMC Evol Biol. 2011 Oct 18;11:307. doi: 10.1186/1471-2148-11-307 (PMC3217942; doi:10.1186/1471-2148-11-307)

# Supplementary material S1

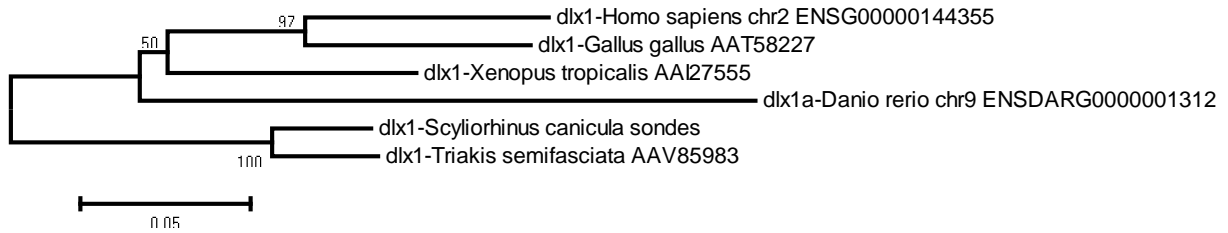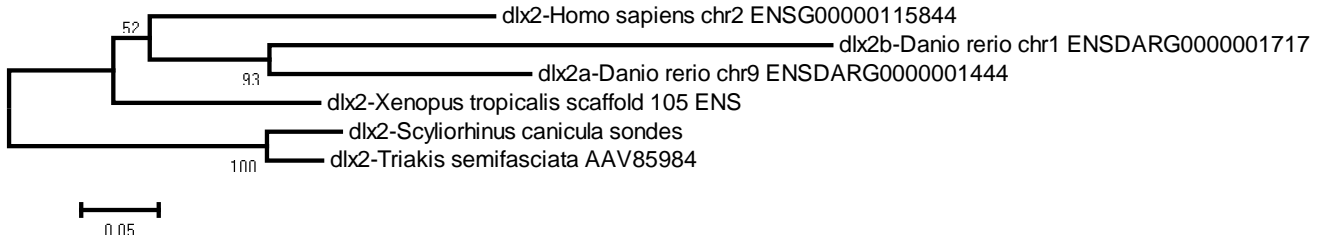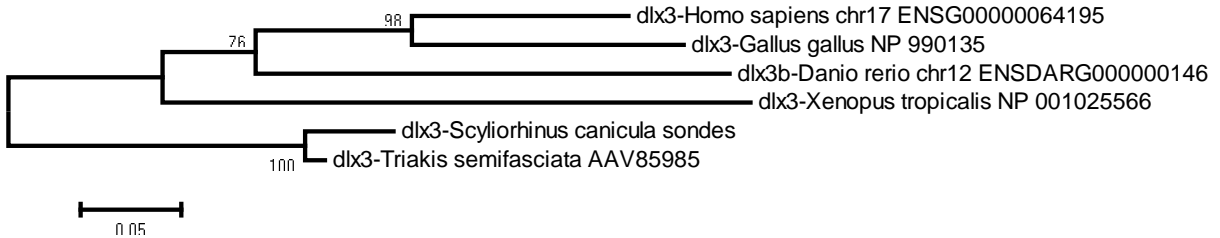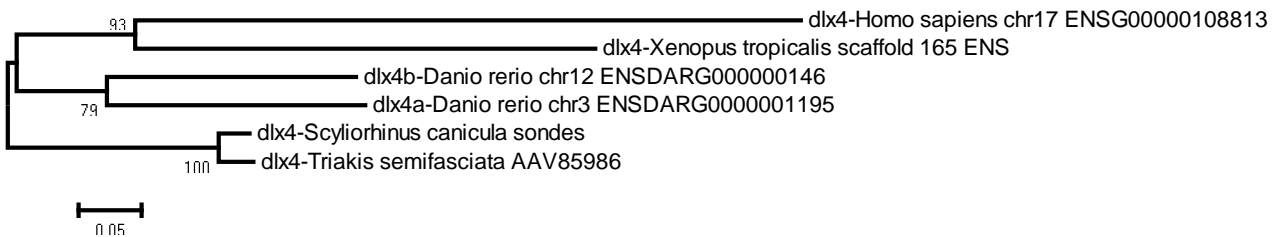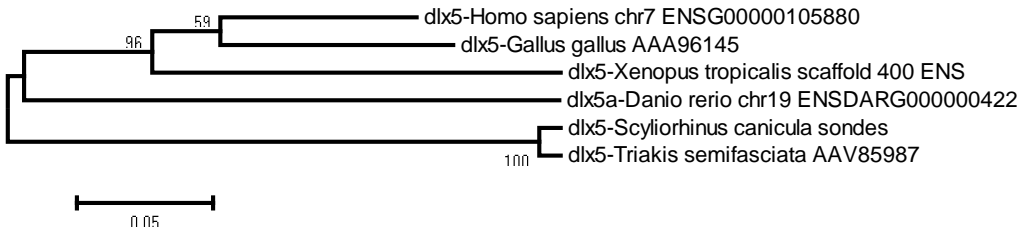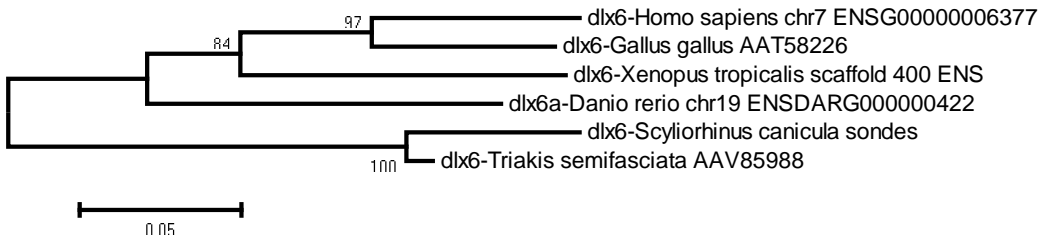

Supplement: Additional file 1 — Assignment of the Dlx probes to their group of orthologues. In order to assign each Dlx probe to an orthologous group, phylogenetic analyses were performed. The Dlx gene sequences of Triakis semifiasciata (leopard shark), Homo sapiens (human), Gallus gallus (chicken), Xenopus tropicalis (xenopus), Danio rerio (zebrafish) were retrieved from Ensembl release 49 and GenBank release 164. Multiple Dlx amino acid sequences were aligned using ClustalX (Thompson et al. 1997) with manual optimization using MUST software (Philippe 1993). Regions of ambiguous homology were removed. Evolutionary distances were computed using the Poisson correction and a neighbor-joining tree was obtained using MEGA4 (Tamura et al. 2007). The robustness of the tree nodes was estimated by a bootstrap test (1000 replicates). Philippe H. 1993. MUST, a computer package of Management Utilities for Sequences and Trees. Nucleic Acids Res 21:5264-5272. Tamura K, Dudley J, Nei M, and Kumar S. 2007. MEGA4: Molecular Evolutionary Genetics Analysis (MEGA) software version 4.0. Mol Biol Evol 24:1596-1599. Thompson JD, Gibson TJ, Plewniak F, Jeanmougin F, and Higgins DG. 1997. The CLUSTAL_X windows interface: flexible strategies for multiple sequence alignment aided by quality analysis tools. Nucleic Acids Res 25:4876-4882. [file 1471-2148-11-307-S1.PDF]

Supplementary material S2

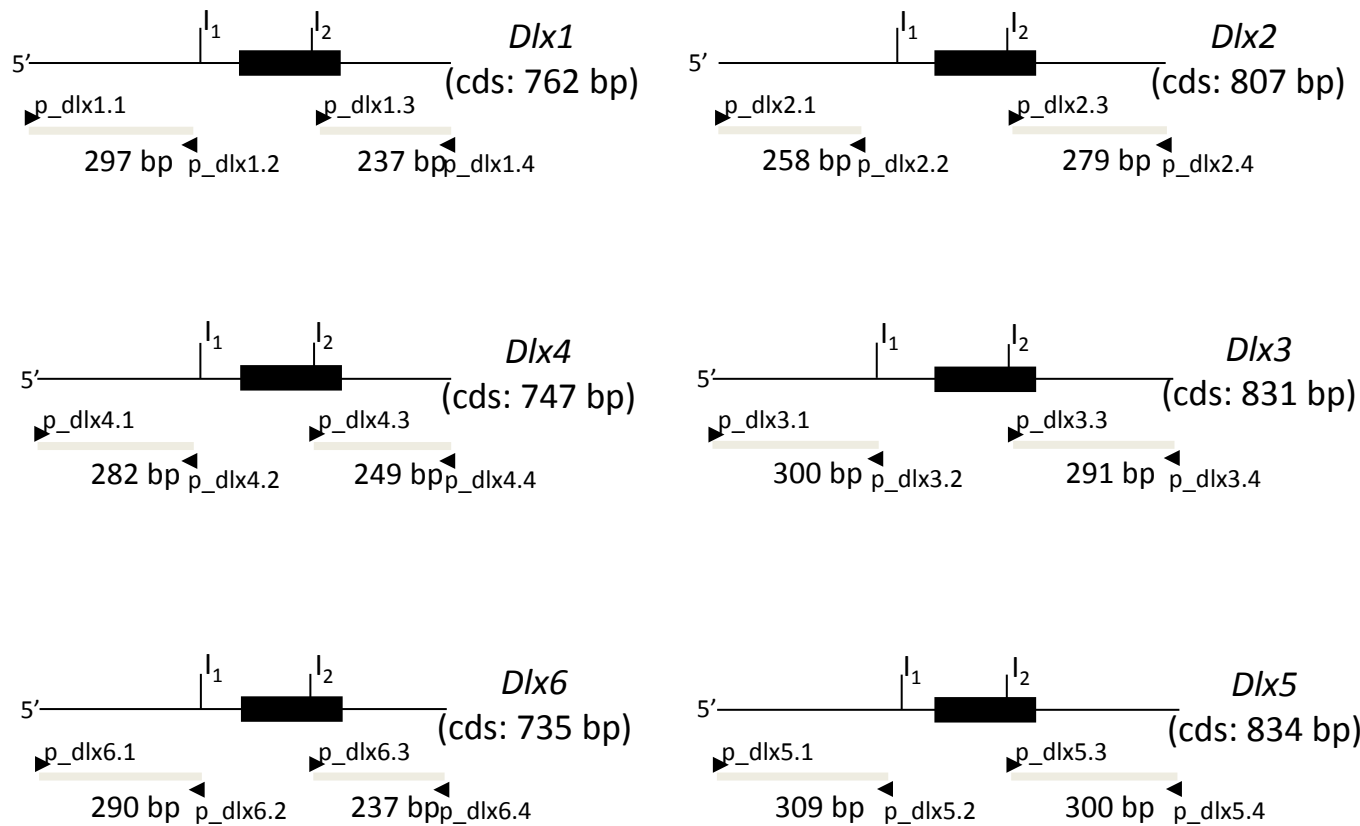

Supplement: Additional file 2 — Position of Dlx probes on the coding sequences of T. semifasciata. The position of a probe is indicated by a grey line. The position of introns (I), the position of homeobox sequences (black box) and the length of each coding sequence (cds) and of each probe are indicated. [file 1471-2148-11-307-S2.PDF]

Supplementary material S3

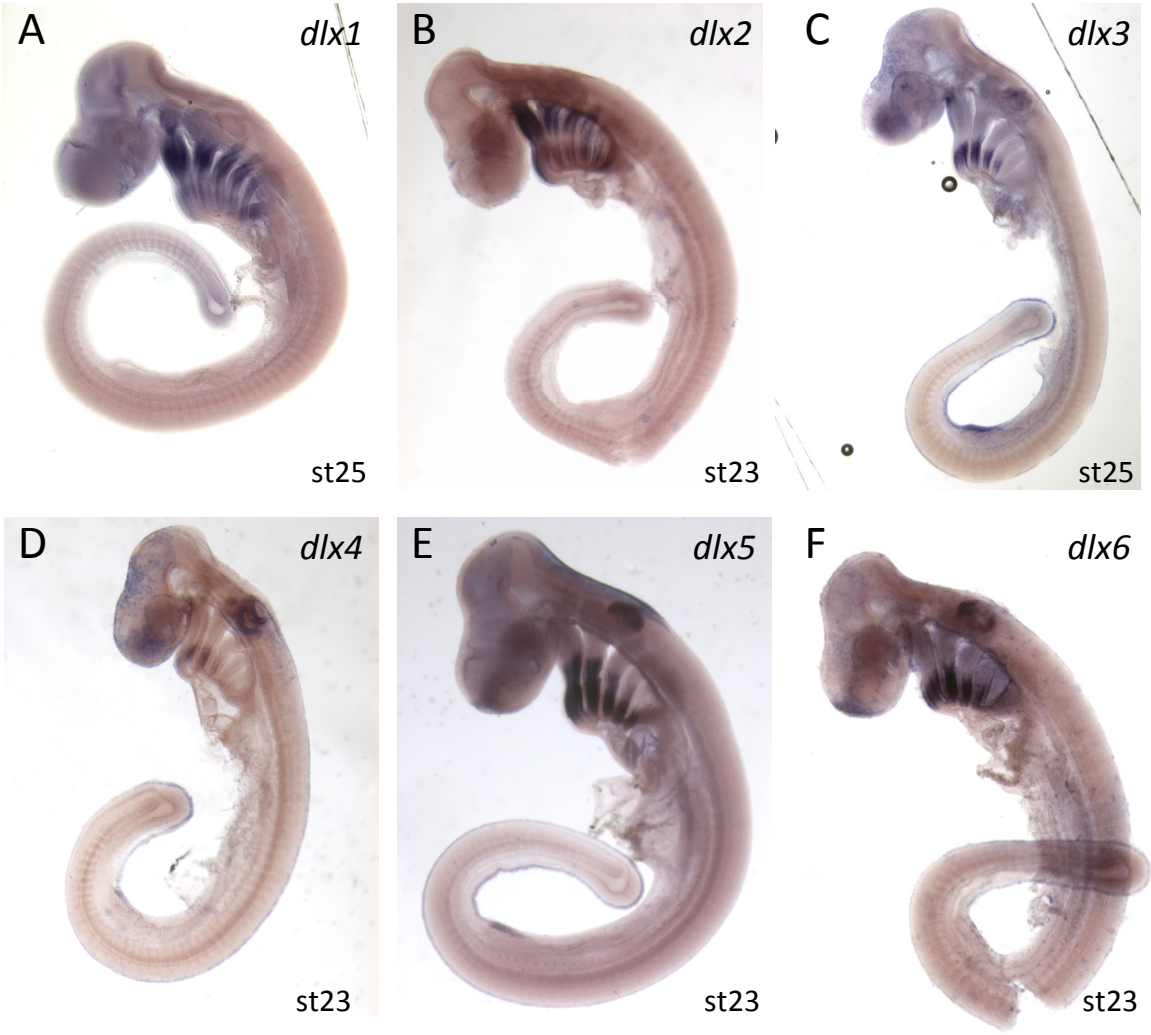

Supplement: Additional file 3 — Test of Dlx probes activity during early stages of embryogenesis in the dogfish. Lateral view of whole mount in situ hybridized embryos with the Dlx probes designed for the 6 dogfish Dlx genes. For each panel, the name of the probe is indicated up right and the stage of the hybridized embryo down right. [file 1471-2148-11-307-S3.PDF]
