# Supplementary material for: The homology of odontodes in gnathostomes: insights from Dlx gene expression in the dogfish, Scyliorhinus canicula
Source: BMC Evol Biol. 2011 Oct 18;11:307. doi: 10.1186/1471-2148-11-307 (PMC3217942; doi:10.1186/1471-2148-11-307)

Supplementary material S4

A.

	medaka-oral	medaka-phar	dogfish-scales	dogfish-teeth	mouse-oral	zebrafish-phar
Dlx1-EM-e	0	0	0	1	0	0
Dlx1-EM-m	0	0	0	0	0	0
Dlx1-LM-e	0	0	1	1	0	0
Dlx1-LM-m	0	0	0	0	1	0
Dlx1-ED-e	0	0	1	1	0	0
Dlx1-ED-m	0	0	1	1	1	0
Dlx1-LD-e	0	0	1	1	0	0
Dlx1-LD-m	0	0	0	0	1	0
Dlx2-EM-e	0	0	1	0	1	1
Dlx2-EM-m	1	1	0	0	1	1
Dlx2-LM-e	0	0	1	0	0	1
Dlx2-LM-m	1	1	1	0	1	1
Dlx2-ED-e	0	0	1	0	0	1
Dlx2-ED-m	1	1	1	0	1	1
Dlx2-LD-e	0	0	1	0	1	1
Dlx2-LD-m	0	0	0	0	0	1
Dlx3-EM-e	1	1	1	1	1	1
Dlx3-EM-m	0	0	0	0	0	0
Dlx3-LM-e	1	1	1	1	0	1
Dlx3-LM-m	1	1	1	1	1	1
Dlx3-ED-e	1	1	1	1	0	1
Dlx3-ED-m	1	1	1	1	1	0
Dlx3-LD-e	1	1	1	1	0	1
Dlx3-LD-m	1	1	0	0	1	0
Dlx4-EM-e	0	0	1	1	0	1
Dlx4-EM-m	0	0	0	0	0	1
Dlx4-LM-e	0	0	1	1	0	1
Dlx4-LM-m	0	0	1	1	1	1
Dlx4-ED-e	0	0	1	1	0	1
Dlx4-ED-m	1	1	1	1	1	1
Dlx4-LD-e	0	0	1	1	0	1
Dlx4-LD-m	1	1	0	0	1	0
Dlx5-EM-e	1	0	1	1	0	1
Dlx5-EM-m	0	0	0	0	0	0
Dlx5-LM-e	1	0	1	1	0	1
Dlx5-LM-m	1	1	1	1	1	1
Dlx5-ED-e	1	1	1	1	0	1
Dlx5-ED-m	1	1	1	1	1	1
Dlx5-LD-e	1	1	1	1	0	1
Dlx5-LD-m	1	1	1	1	1	1
Dlx6-EM-e	0	0	0	0	0	0
Dlx6-EM-m	0	0	0	0	0	0
Dlx6-LM-e	0	0	0	0	0	0
Dlx6-LM-m	0	0	0	0	1	0
Dlx6-ED-e	0	0	0	0	0	0
Dlx6-ED-m	1	1	0	0	1	0
Dlx6-LD-e	0	0	0	0	0	0
Dlx6-LD-m	1	1	0	0	1	0

Supplementary material S4

B.

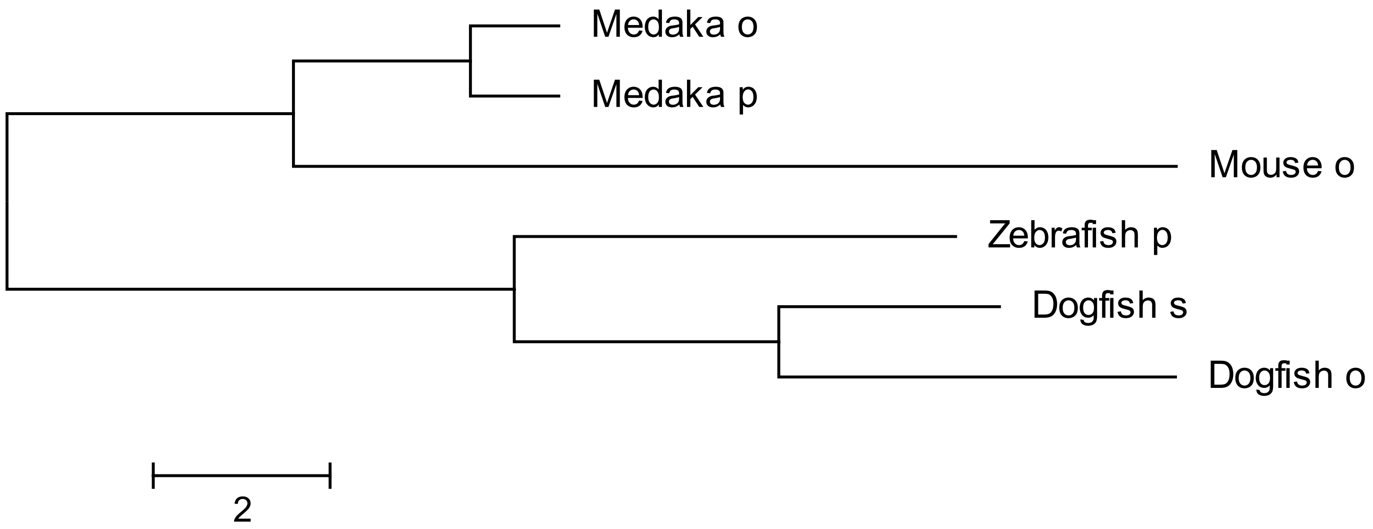

Supplement: Additional file 4 — Statistical evaluation of differences between Dlx expression patterns in gnathostome odontode development. A. Matrix describing the expression pattern of each Dlx gene from the mouse, zebrafish, medaka and dogfish, during all four odontode developmental stages (see text for a description), either in the epithelial (e) or mesenchymal (m) compartment. Data are as of Table 3, but different columns were made for oral (o) versus pharyngeal (p) teeth in medaka, or scales (s) versus oral teeth (o) in dogfish. B. Neighbor Joining tree inferred with a pairwise distance (number of differences) matrix estimated with the matrix of characters shown in A. [file 1471-2148-11-307-S4.PDF]
