# Supplementary material for: The homology of odontodes in gnathostomes: insights from Dlx gene expression in the dogfish, Scyliorhinus canicula
Source: BMC Evol Biol. 2011 Oct 18;11:307. doi: 10.1186/1471-2148-11-307 (PMC3217942; doi:10.1186/1471-2148-11-307)
Supplement: Additional file 5 — Primer sequences. Primer names follow the description in the Additional file 2. [file 1471-2148-11-307-S5.PDF]

Supplementary material S5

p_dlx1.1	5'-	ATGACCATGACYRCGATGYC	-3'
p_dlx1.2	5'-	CCGCGTATGGACNAGRCTNGA	-3'
p_dlx1.3	5'-	GTNAAAATNTGGTTYCAGAA	-3'
p_dlx1.4	5'-	CTGCTGCATAGAYTAYTGRTG	-3'
p_dlx2.1	5'-	ATGACYGGNGTGTTYGAYA	-3'
p_dlx2.2	5'-	GGAATAACTAATYTCRTANGAYTT	-3'
p_dlx2.3	5'-	GTAAARATNTGGTTCCAGAA	-3'
p_dlx2.4	5'-	GGGCTGATGTTGAAGNATNGG	-3'
p_dlx3.1	5'-	ATGAGYGGAACYTAYGANAA	-3'
p_dlx3.2	5'-	GAATGCCCRTAYTGNCKRTA	-3'
p_dlx3.3	5'-	GTGAARATYTGGTTYCAGAA	-3'
p_dlx3.4	5'-	AGTACTCTGAGGTGGTGGYTGRTG	-3'
p_dlx4.1	5'-	ACCGCGATGGCTGACGGRYTRTT	-3'
p_dlx4.2	5'-	CTCTAACCTGGTGTGACCCARNGT	-3'
p_dlx4.3	5'-	GTNAAAATNTGGTTYCAGAA	-3'
p_dlx4.4	5'-	CATCATYTGRGKYCTNGSCAT	-3'
p_dlx5.1	5'-	ACAGGAGTATTCGATCGYAARYT	-3'
p_dlx5.2	5'-	TGGCTGAGCCCTGCTRTANGC	-3'
p_dlx5.3	5'-	GTNAAAATNTGGTTYCAGAA	-3'
p_dlx5.4	5'-	GGATCCCARNGCYAGNGGRTG	-3'
p_dlx6.1	5'-	ATGACCATGACYACNATGG	-3'
p_dlx6.2	5'-	GTCTCGTCTAANCGNGTYTG	-3'
p_dlx6.3	5'-	GTRAAGATYTGGTTYCARAA	-3'
p_dlx6.4	5'-	TCTYTGCACNGTRTCYTGRTG	-3'
